# Evaluating the impact of the single exit price policy on a basket of originator medicines in South Africa from 1999 to 2014 using a time series analysis

**DOI:** 10.1186/s12913-019-4403-8

**Published:** 2019-08-16

**Authors:** R. Moodley, F. Suleman

**Affiliations:** 1 Discipline of Pharmaceutical Sciences, School of Health Sciences, Westville Campus, University of KwaZulu-Natal, Private Bag X54001, Durban, 4000 South Africa; 2 Prince Claus Chair of Development and Equity for the theme Affordable (Bio) Therapeutics for Public Health (September 2016 to September 2018), Faculty of Sciences, Utrecht University, Utrecht, The Netherlands

**Keywords:** Single exit Price, South Africa, Time series, Medicine pricing policy

## Abstract

**Background:**

Affordability and availability of quality medicines to all its citizens has been a key priority area for South Africa since democracy in 1994. In order to introduce transparency in the private market the government introduced the Single Exit Price (SEP) for medicines in 2004, for all prescription medicines, comprising of a fixed ex-factory price with a logistics fee component (and value added tax) for medicines sold to all purchasers other than the State. This is complemented with a provision for an annual regulated maximum percentage increase. The study evaluates the impact of the SEP on a basket of originator medicines, in terms of costs, immediate price reductions and projected price reductions.

**Method:**

This is an analytical, quantitative study. A basket of medicines was selected, based on the WHO/HAI list, and adapted to include registered medicines in South Africa. Prices of 50 originator medicines were assessed from 1999 to 2014 in terms of the single exit price and the changes in prices in accordance with legislation using a time series analysis methodology.

**Results:**

Of the 50 originator medicines investigated 35 showed a statistically significant change in level. For the Global Core list, the percentage change ranged from 2.45–39.12% (mean = 19.87%, SD = 10.62%, IQR = 10.2%). The range for the Regional Core list was 1.77–42.17% (mean = 23.38%, SD = 12.43%, IQR = 15.65%). The Supplementary list was 11.68–55.86% (mean = 22.97%, SD = 16.26%, IQR = 17.34). This study indicates that the SEP regulation had an impact on medicine pricing in South Africa in both the short and long term. Most medicines investigated showed a smaller yearly increase in price compared to before regulations due to the controlled pricing environment introduced by Government.

**Conclusion:**

This study provides evidence of the impact of medicine pricing intervention from a middle–income country, and other developing countries looking at introducing medicine price controls can draw useful lessons.

## Background

The complex nature of any country’s pharmaceutical supply chain makes it extremely sensitive to medicine pricing policy changes [1] especially in low to middle-income countries (LMICs). It is therefore important that when change does occur the impact of the change is measured.

Growing expenditure on pharmaceuticals in both the public and private sector in many parts of the world has been a source of concern for healthcare professionals, patients, funders and Governments alike. The per capita spending in pharmaceuticals [2], as per the National Health Accounts (NHA) reports increased by approximately 50% (*n* = 135–148 countries) between 1995 and 2006. Medicine spend [3] in low and middle-income countries accounts for 20–60% of the health care budgets. Further to this, the World Health Organisation (WHO) [3] estimated that 90% of peoplein developing countries buy medicines through out of pocket payments, resulting in this being the second largest family spend next to food.

Many governments have thus introduced pricing interventions to reduce medicine prices for payers and patients alike, but very little evidence exists as to their impact. Moreno-Torres [4] analysed sixteen interventions introduced to control the pharmaceutical expenditure in Spain and found that twelve interventions were not effective in decreasing medicine prices even in the short term, and the other four interventions did not have sustained impact in the long term resulting in a moderate annual saving.

Sood et.al [5] in describing policy interventions in nineteen developed countries from 1992 to 2004, found that cost reduction effects of price control increased the longer they remained in effect. The authors further concluded that introducing new policies in an unregulated market [6], such as the United States (US) could greatly reduce pharmaceutical spending. If the US did introduce pricing policies it is projected that prices of medicines could fall by 20.3% [5].

Carone et al. [7] suggests that regulating pharmaceutical markets “comes as an answer to classic market failures of healthcare markets”. Most European Union member states (*n* = 24), set their prices through external reference pricing (ERP -establishing a price on the basis of price of the same product in other countries) while some countries use an internal reference price (IRP) where prices are based on market equivalent or similar products within the country [8].

Other low and middle-income countries have introduced pricing policies to manage medicine prices. Brazil in 1998 through its Federal Government implemented the Banco de Prescos em Saude (BPS) to facilitate a transparent measure that centralized the pricing information [9]. Argentina has a mandatory report of purchase price policy. Schargrodsky et al. [10] analysed the mandatory report of purchase price in 33 hospitals in Buenos Aires. The results confirmed that medicine prices significantly decreased after the mandatory policy, but this was not sustained, and prices eventually increased over time [10]; an indication that mandatory reporting and publishing medicine prices as a policy is insufficient to impact on medicine price reduction.

Ecuador in 2014 [11] introduced price control for essential medicine which accounted for 54% of their pharmaceutical market. Colombia in 2011 introduced a compulsory cap on inpatient drug reimbursement by active ingredient, and in 2013 introduced an ERP using the markets in 17 countries and further regulated prices set at the 25 percentile. A study by Prada et al. [12] suggested that after implementation of direct price control there was a 43% decrease in price inflation, but expenditure doubled due to the disproportunate increase in units sold.

Many of these examples in the South American region illustrate the government efforts to improve transparency in pricing and procurement [9]. Kohler et.al [9] concluded that pricing transparency should allow for decrease in medicine prices, but other measures are required to ensure sustainability of price optimization.

In terms of pricing regulations within the African context, Sudan introduced a National Health Insurance Fund (NHIF) in 1995 and achieved national cover by 2010 [13]. Medicine expenditure between 2006 and 2010 in Sudan grew at an annual rate of 35.78%. This was assumed to be the direct result of increased utilization related to the greater coverage. Mousnad and colleagues [13] further defined other multiple factors contributing to price increases, including the global economic crisis, increased government taxes, custom and clearance duties, and price increases in the exporting countries.

Nguyen et al. [1] suggest that there is sufficient evidence to show that high-income countries are using a variety of pricing and purchasing methods to contain pharmaceutical expenditure. In low income countries with more than half and sometimes up to 90% of out-of-pocket expenditure on medicines ([14], it has not been easy to implement pricing policies.

### South Africa’s policy changes

South Africa experienced similar issues in terms of increasing medicine costs and expenditure. Data from Council for Medical Schemes (CMS) in South Africa indicated that medicine expenditure was the main cost driver in the 1980’s and early 1990’s peaking at 31.8% of the total medical scheme spend in 1993 [15].

The South African Governments pre-1994, led several attempts to regulate the medicine-pricing environment, in terms of changes to the Medicines and Related Substances Act [16], primarily in Section 18A and Section 22G [17]. These changes attempted to introduce a transparent pricing system by firstly ensuring that there was a Single Exit Price (SEP) for all medicines sold by the manufacturers to all distributors/dispensers in the country. The SEP is set by the manufacturer, and covers all medicines registered in South Africa. Exemptions have been provided to over-the-counter medicines (schedule zero medicines in South Africa) and veterinary medicines. The policy thus applies to all prescription medicines in the private sector. The SEP is composed of the ex-manufacturer price (as determined by the manufacturer), the logistic fee (as determined by the manufacturer) and the value added tax component (14%) for these medicines sold to all purchasers other than the State. This is complemented with a provision for a regulated maximum percentage increase in the single exit price, determined annually by the Minister of Health, on the recommendation of the Pricing Committee. This was combined with the removal of all bonuses, discounts and sampling of medicines (Section 18A).

The only published study on medicine pricing in South Africa, was done in December 2004 [18], that highlighted the issue of medicine prices in the Gauteng Province. The study utilized a similar methodology as outlined by WHO and Health Action International (HAI) [19] but utilized data primarily from the period before the full implementation of the SEP. The authors recommended in their conclusion that further studies be conducted to include all provinces in the country after full implementation of the SEP.

With regulatory changes showing different outcomes in various parts of the world [4] it is critical that the impact of these interventions in South Africa be measured. Evidence is needed to determine firstly, if the legislative changes did achieve the intended outcomes and secondly to give guidance to policy makers regarding any national and institutional problems that may have arisen as an unintended consequence. For South Africa in particular, this study may form an important tool in determining pricing strategies in the new National Health Insurance (NHI) and Universal Health Coverage (UHC).

There has been some research conducted on medicine expenditure post SEP implementation. A substantial decrease occurred between 2004 and 2005 [20]. The authors estimated that the SEP changes contributed to a 22% decrease in the average prices of medicines.

The Mediscor Medicines Review 2004 [16] suggested that various parties believed that the SEP regulations reduced medicine prices by between 18 and 19% translating to a R2.5 billion reduction in the industry turnover. From January 2004 to August of the same year Mediscor experienced a 19% decrease in medicine SEP, viz. a 14% reduction in branded products and 35% in generic equivalents [16] . The top 5 classes of medicines decreased in SEP as follows, cardio vascular agents 12%, central nervous system agents 16.3%, antimicrobials 25.9%, endocrine agents 15.5% and respiratory system agents by 27.3%.

The National Department of Health reported a 19% average reduction of SEP in 2004 with a 25–30% reduction in generic medicines and a 12% reduction in originators prices [17]. Medscheme in their submission to the Market Health Inquiry [21] indicated that annual SEP increases since the introduction of the regulation in 2004 fell mostly below Consumer Price Index increases and on a typical basket of medicines the average price increase fell below the published SEP increases [21].

However, no focused research has been conducted on the impact of the SEP policy on medicine prices, to ascertain whether actual sustained price reductions were achieved. This paper thus tries to address this gap by evaluating the impact of SEP on a basket of originator medicines, in terms of costs, and impact on prices.

## Methods

A quantitative analytical approach was used in this study. The setting was the South African private sector, as the SEP regulation did not apply to the state sector where medicines are largely acquired via a tender system. The study was granted ethical clearance by the University of KwaZulu-Natal Human and Social Sciences Research Ethics Committee (HSS/0154/013). In looking at the impact of legislative changes on prices, a longitudinal method [22] for pharmaceutical policy evaluation was used with the specific application of the interrupted-time series (ITS). Longitudinal trends were compared before and after the introduction of policy changes. The research tracked annual price changes on a basket of products five years before regulatory changes and then measured annual SEP changes over the next ten years, following the intervention, viz. from 1999 to 2014.

The changes in medicine prices over a specified period prior to 2004 formed the time series i.e. a sequence of medicine prices over a range of medicines taken at a regular spaced interval – prices registered in December of each year (when there were no more price changes in the system). The time of the regulatory introduction formed the change point. This is the specific points in time where the values of the time series should exhibit a change from previous established pattern, in this case a regulatory or policy change.

Commonly used data source for time series is cost data obtained from pharmacy dispensing files, claims data, and other routinely collected data. SEPs of medicines listed were obtained from the computer vendors responsible for maintaining price files for pharmacy and verified through the pharmacy dispensing systems spanning the period 1999 to 2014. The Government medicine price database [23], was created after the introduction of the SEP, and only exists post the intervention and therefore could not be utilized. It was also important to utilize a single complete data source to ensure accuracy of results.

Pricing data for the medicines being studied could not be obtained before 1999 in the country and was identified as a limiting factor. Stata (13 MSI), a statistical package was used to analyse the data, generate the necessary variables, compute the statistical analysis and produce the necessary graphs [24].

### Selection of the basket of products

A basket of fifty (50) medicines were chosen implementing the World Health Organisation/Health Action International (WHO/HAI) [25] recommendation. This was to ensure that our research measuring medicine prices was in keeping with the international methodology currently being applied in more than 50 countries [25]. Utilizing these standard guidelines also allows us to contribute to the research evidence classified by WHO/HAI as ‘scarce’ in low-and middle-income countries [25].

The Global Core of fourteen items (14) allows for international comparison, a Regional Core of fifteen (15) items allows for regional differences in medicine usage whilst still enabling comparison across countries and the twenty-one (21) medicines from a supplementary list selected for their local importance [25] completed the basket. While the May 2016 update on the WHO/HAI [14] recommendation indicate a removal of the Regional Core in favour of 36 medicines chosen by the national investigator, this study used the original recommendation since the investigation spanned the 1999 to 2014 period. Further, since the regulations affected mainly the private sector in South Africa, an assessment of the top 50 medicines dispensed (by volume) in the private sector (IMS Health) in 2014 was taken into consideration. This data was sourced from IMS Health and used in the supplementary list. Consideration was also given to the list used in the 2004 study [18] for further comparison. Once the 50 medicines were selected, the originator product was listed together with the strength, form, pack-size and National Pharmaceutical Product Index (NAPPI). The NAPPI code is a unique coding system used in South Africa. This allowed ease of reference when pricing was compared from different data files. Any price change listed on the data file in December of each year was captured.

## Results

Tables 1, 2 and 3 below represents the results of the interrupted time-series analysis (ITSA) for three groups of fifty (50) originator medicines listed as Global Core, Regional Core and Supplementary respectively. The global core in Table 1 contains the data for 14 originator molecules. Of the fourteen (14) original molecules ten (10) showed a statistically significant (*P* < 0.05) change in level. The level change indicated an immediate decrease in the medicine price on the introduction of the regulation in 2004. 71.43% of the molecules showed a statistically significant (*P* < 0.05) change in slope indicating that the policy will continue to benefit medicine prices over time.

**Table 1:** Interrupted time-series analysis for originator molecules in the global core list, using pricing data from 1999 to 2014 with 2004 as the interruption in the series (*P* < 0,05)

INN		Trend	(*P* value)	Change in level	(*P* value)	Change in slope	(*P* value)	Constant	(*P* value)	Int 1	*% Change in level 2004*
Salbutamol 2 mg/5mls Syr	1, Ventolin	0,018	0,000	−0,065	0,000	−0,014	0,000	0,19	0,000	0,28	−23,47
Glibenclamide 5 mg tab	2, Daonil	0,228	0,000	−0,771	0,001	−0,047	0,382	2,45	0,000	3,59	−21,51
Atenolol 50 mg caps	3, Tenormin	0,427	0,000	−1242	0,000	−0,209	0,007	2,56	0,000	4,70	−26,45
Captopril 25 mg tabs	4, Capoten^a^	0,044	0,014	−0,117	0,071	0,016	0,365	2,09	0,000	2,31	−5,07
Simvastatin 20 mg tabs	5, Zocor	−0,997	0,001	− 1078	0,225	0,832	0,004	9,56	0,000	4,58	−23,54
Amitriptyline 25 mg tabs	6, Tryptanol	0,176	0,000	−0,397	0,000	−0,169	0,000	1,70	0,000	2,58	−15,42
Ciprofloxacin 500 mg tabs	7, Ciprobay	−1028	0,002	−5113	0,000	1560	0,000	18,21	0,000	13,07	−39,12
Co-Trimoxazole 8 + 40 mg/ml syr	8, Bactrim	0,364	0,000	−2267	0,000	−0,247	0,000	2,46	0,000	4,28	−52,94
Amoxicillin 500 mg caps	9, Amoxil^b^	0,334	0,000	−0,127	0,429	−0,274	0,001	3,52	0,000	5,19	−2,45
Ceftriaxone 1 g/vial inj	10, Rocephin	4302	0,081	−82,503	0,000	−3371	0,237	121,81	0,000	143,32	−57,57
Diazepam 5 mg	11, Valium	0,318	0,000	−0,772	0,000	−0,213	0,000	1,00	0,000	2,59	−29,83
Diclofenac 50 mg tabs	12, Voltaren	0,063	0,013	−0,209	0,025	0,021	0,373	1,16	0,000	1,48	−14,17
Paracetamol 25 mg/ml syr	13, Panado	0,001	0,702	−0,030	0,017	0,014	0,000	0,18	0,000	0,18	−16,57
Omeprazole 20 mg tabs	14, Losec	−0,610	0,036	1183	0,245	1298	0,000	11,58	0,000	8,53	13,87

Withdrawn- ^a^2009 ^b^ 2008

Each item carries the® for trademark reference

**Table 2:** Interrupted time-series analysis for originator molecules in the regional core list, using pricing data from 1999 to 2014 with 2004 as the interruption in the series. Statistically significant values (*P* < 0,05)

INN		Trend	(*P* value)	Change in level	(*P* value)	Change in slope	(*P* value)	Constant	(*P* value)	Int 1	*% Change in level 2004*
Albendazole 200 mg tabs	15, Zentel^a^	0,571	0,002	−2812	0,000	0,740	0,001	12,272	0,000	15,127	−18,59
Amlodipine 5 mg Tabs (99,100,101)^g^	16, Norvasc	0,305	0,082	−2447	0,002	−0,201	0,254	4278	0,000	5803	−42,17
Atovastatin 20 mg Tabs (102,103,104)^g^	17, Lipitor	0,349	0,001	−2645	0,000	−0,114	0,170	7665	0,000	9,41	−28,11
Beclomethasone100mcg/dose inh	18, Becotide^b^	−17,698	0,035	− 6847	0,809	18,412	0,269	164,637	0,000	76,147	−8,99
Cephalexin 250 mg caps	19, Keflex^c^	0,78	0,004	−7919	0,000	−0,752	0,093	5665	0,000	9565	−82,79
Enalapril 10 mg tabs	20, Renitec	−0,56	0,000	0,159	0,589	0,573	0,000	3859	0,000	1059	15,01
Fluoxetine 20 mg tabs	21, Prozac	0,579	0,000	−2787	0,000	−0,324	0,001	6021	0,000	8916	−31,26
Gliclazide 80 mg tabs	22, Diamicron^d^	0,093	0,004	−0,311	0,010	−0,055	0,084	0,873	0,000	1338	−23,24
Hydrochlorothiazide 25 mg tabs	23, Dichloride^e^	0,031	0,178					0,742	0,009	0,897	0,00
Ibuprofen 200 mg tabs	24, Brufen^f^	0,034	0,000	−0,103	0,000	−0,019	0,006	0,419	0,000	0,589	−17,49
Metformin 500 mg tabs	25, Glucophage	−0,021	0,027	−0,2	0,000	0,038	0,001	0,606	0,000	0,501	−39,92
Metronidazole 200 mg tabs	26, Flagyl	0,195	0,000	−0,609	0,000	−0,125	0,000	0,721	0,000	1696	−35,91
Nifedipine Retard 10 mg tab	27, Adalat Ret	0,324	0,000	−0,632	0,003	−0,147	0,016	1788	0,000	3408	−18,54
Ranitidine 150 mg tabs	28, Zantac	0,333	0,005	−0,101	0,777	0,024	0,824	4038	0,000	5703	−1,77
Sodium Valproate200mg Tab	29, Epilim	0,151	0,000	−0,307	0,016	−0,035	0,292	1344	0,000	2099	−14,63

Withdrawn ^a^ 2010 ^b^ 2006 ^c^2006 ^d^ 2009 ^e^2001 ^f^ 2009

Each item carries the® for trademark reference

^g^number for molecules with no data in the list

**Table 3:** Interrupted time-series analysis for originator molecules in the supplementary .list, using pricing data from 1999 to 2014 with 2004 as the interruption in the series. Statistically significant values (*P* < 0,05)

INN		Trend	(*P* value)	Change in level	(*P* value)	Change in slope	(*P* value)	Constant	(*P* value)	Int 1	*% Change in level 2004*
Acyclovir 200 mg tabs	30, Zovirax	0,677	0,000	−0,429	0,422	−0,2	0,187	8551	0,000	11,936	−3,59
Carbamazepine 200 mg tabs	31, Tegretol	0,175	0,000	−0,477	0,001	−0,078	0,023	1464	0,000	2339	−20,39
Amox/Clav inj 600 mg (146,147)^a^	32, Augmentin	3,13	0,000	−9,99	0,001	−2211	0,006	7841	0,000	23,491	−42,53
Digoxin 0,25 mg tab	33, Lanoxin	0,021	0,000	−0,09	0,000	−0,014	0,001	0,206	0,000	0,311	−28,94
Fluconazole 200 mg cap (149, 150, 151)^a^	34, Diflucan	−0,238	0,729	−6626	0,013	2738	0,004	50,703	0,000	49,513	−13,38
Ketoconazole 200 mg Tab (153)^a^	35, Nizoral	2079	0,000	−3,45	0,017	−1107	0,031	13,609	0,000	24,004	−14,37
Losartan 50 mg Tab (154, 155)^a^	36, Cozaar	−0,017	0,948	− 0,096	0,911	− 0,276	0,381	5213	0,000	5128	−1,87
Phenytoin 100 mg caps (156)^a^	37, Epanutin	0,13	0,000	−0,43	0,000	−0,055	0,028	0,979	0,000	1629	−26,40
Rifampicin 150 mg caps (157)^a^	38, Rimactane	0,185	0,000	−0,555	0,000	−0,132	0,000	0,542	0,000	1467	−37,83
Rosuvastatin 10 mg Tabs (158, 159, 160)^a^	39, Crestor (no data)	0,239	0,000					4551	0,000	5746	0,00
Ofloxacin 200 mg Tabs (162)^a^	40, Tarivid	2128	0,000	−5167	0,000	−1113	0,004	10,353	0,000	20,993	−24,61
Aminophylline 250 mg inj	41, Aminophylline	2178	0,000	−7891	0,000	−0,944	0,040	16,003	0,000	26,893	−29,34
Miconazole Nitrate 2% crm (166)^a^	42, Daktarin	0,526	0,000	−0,971	0,001	−0,269	0,003	2,4	0,000	5,03	−19,30
Erythromycin 250 mg tabs	43, Erythrocin	0,329	0,323	0,7	0,611	−1915	0,004	4346	0,000	5991	11,68
Azithromycin 500 mg Tabs (169, 170, 171)^a^	44, Zithromax	2872	0,000	−10,698	0,000	−1595	0,017	27,625	0,000	41,985	−25,48
Cimetidine 200 mg tabs	45, Lenamet	−0,22	0,000	−0,31	0,001	−0,238	0,000	1655	0,000	0,555	−55,86
Lisinopril 10 mg Tabs (175, 177)^a^	46, Zestril	0,187	0,231	−1,66	0,231	−0,217	0,186	2917	0,000	3852	−43,09
Loratadine 10 mg Tabs (178, 179, 180)^a^	47, Clarityne	0,249	0,267	− 1013	0,230	−0,667	0,012	5342	0,000	6587	−15,38
Ceftazidime I1g/vial inj (181, 182, 183)^a^	48, Fortum	10,593	0,000	−35,972	0,000	−7382	0,004	98,727	0,000	151,692	−23,71
Isosorbide Mononitrate 20mgT	49, Ismo	0,377	0,000	−1195	0,000	−0,255	0,000	1153	0,000	3038	−39,34
Thyroxine 50mcg Tab (185, 186)^a^	50, Eltroxin	0,034	0,001	−0,028	0,303	−0,004	0,614	0,333	0,000	0,503	−5,57

Each item carries the® for trademark reference

Crestor- no pre- date available

^a^number for molecules with no data in the list

Table 2 contains the data for the regional core basket of 15 original medicines. Of the 15 originator molecules 11 showed a statistically significant change in level (*P* < 0.05) with 7 showing statistically significant change in slope.

In the 21 molecules (Table 3) analysed in the supplementary basket 14 showed statistically significant change in level (66.67%) and 16 (76.19%) showed statistically significant change in slope (*P* < 0.05).

The following formula was used to calculate the limits used to define outliers in the data set for each of the three categories:

$$ \mathrm{Upper}\kern0.5em \mathrm{limit}:\kern0.5em \mathrm{Q}3+\left(\mathrm{IQR}\times 1.5\right) $$

$$ \mathrm{Lower}\kern0.5em \mathrm{limit}:\kern0.5em \mathrm{Q}1\hbox{-} \left(\mathrm{IQR}\times 1.5\right) $$

Anything outside of the calculated limits was identified as an outlier and excluded from the data set. Once the outliers were excluded, descriptive statistics were performed on the three data sets including calculations of the mean, standard deviation, and inter-quartile range (IQR). The descriptive statistics are presented in boxplot below.

**Fig. 1:**
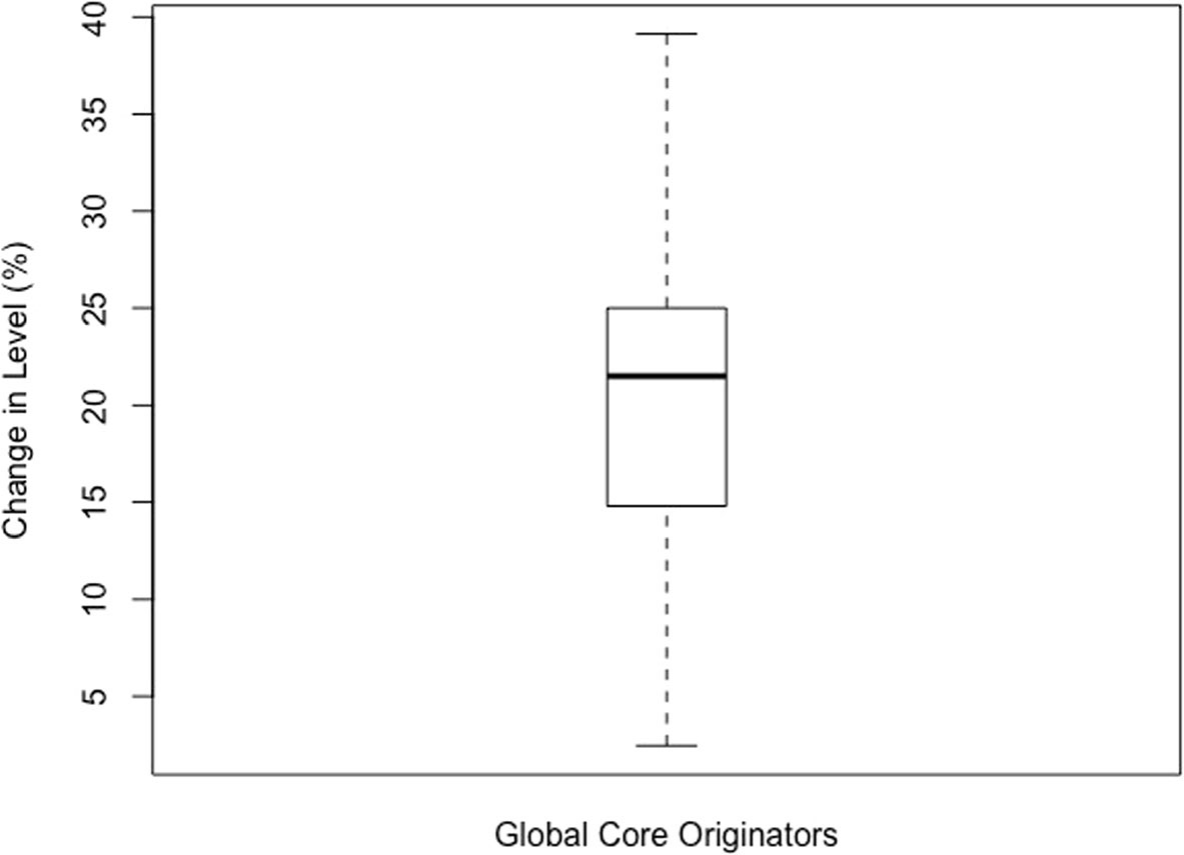
Percentage change in level in the Global Core basket

**Fig. 2:**
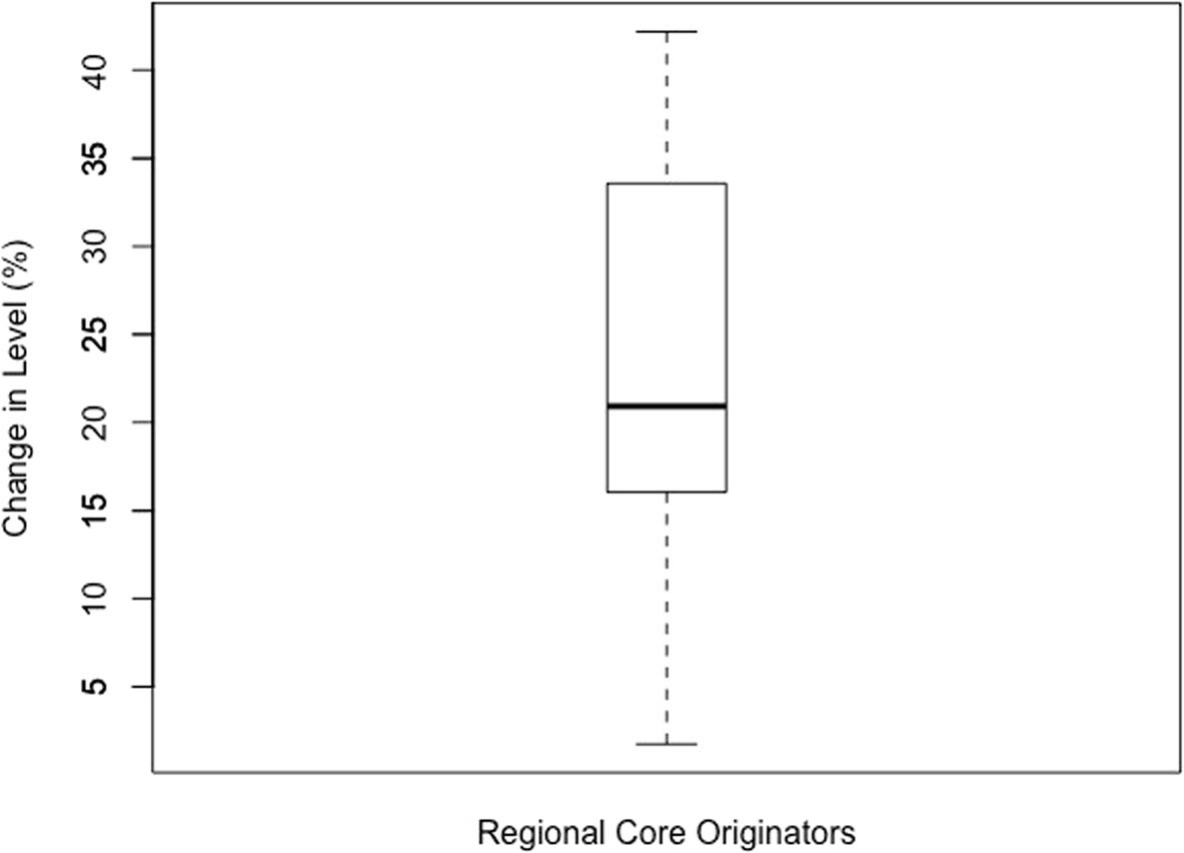
Percentage change in level in the Regional Core basket

**Fig. 3:**
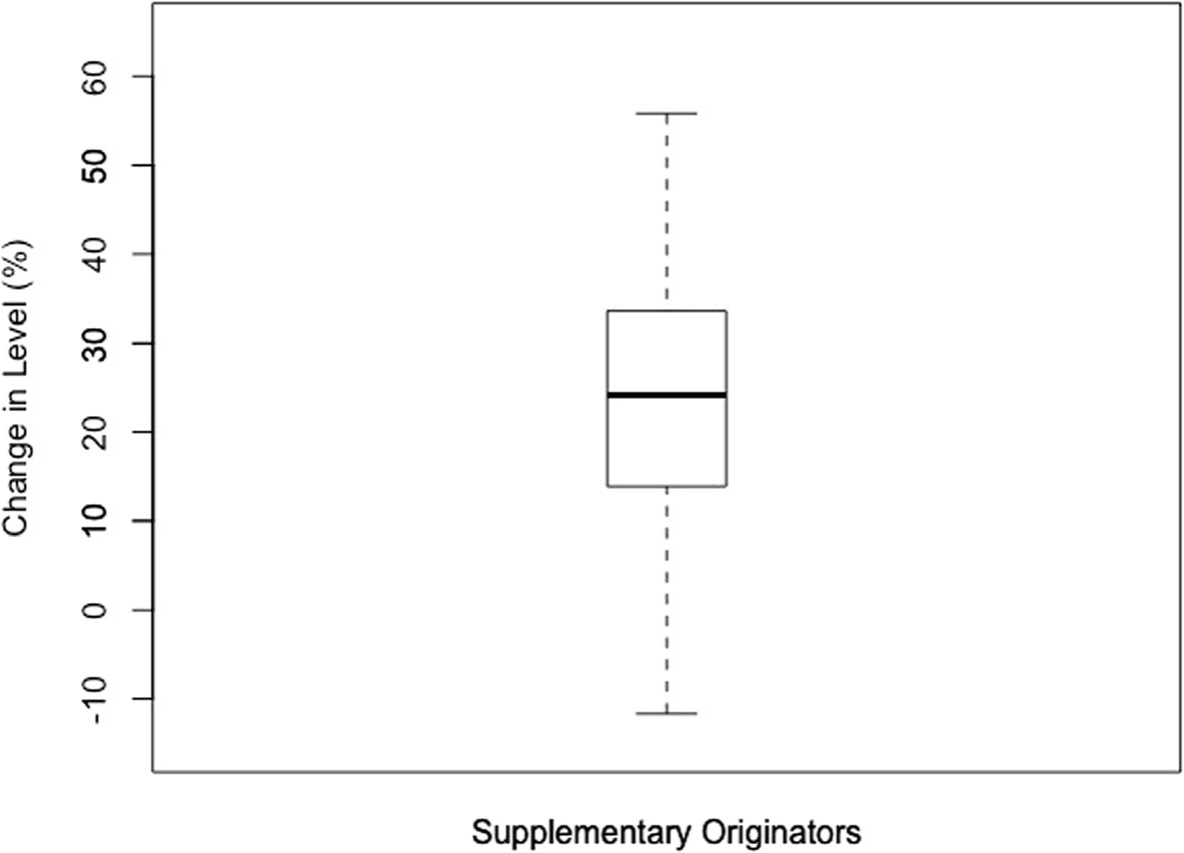
Percentage change in level in the Supplementary basket

The boxplots of percentage change in level for each category of medicines are reflected below. For the Global Core (Fig. 1) the percentage change ranged from 2.45–39.12% (mean = 19.87%, SD = 10.62%, IQR = 10.2%). The range for the Regional Core (Fig. 2) was 1.77–42.17% (mean = 23.38%, SD = 12.43%, IQR = 15.65%). The Supplementary list (Fig. 3) was 11.68–55.86% (mean = 22.97%, SD = 16.26%, IQR = 17.34). The negative values in the minimum reflects an increase in price (positive change in level), and all calculations excludes outliers.

Three trends emerged from all the medicines examined (see Table 4). These trends are further explained in the text that follows.

**Table 4:** Emerging trends of originator medicines

	Trend 1	Trend 2	Trend 3
Global Core	1. Ventolin®	5. Zocor	4. Capoten
	2. Daonil®	7. Ciprobay	6. Tryptanol
	3. Tenormin	14. Losec	9. Amoxil
	8. Bactrim		
	10. Rocephin		
	11. Valium		
	12. Voltaren		
	13. Panado		
Regional Core	16. Norvasc	25 Glucophage	15 Albendazole^a^
	17. Lipitor	20 Renitec	18 Becotide ^a^
	21. Prozac		19 Keflex
	26. Flagyl		22 Diamicron
	27. Adalat Retard		23 Dichloride
	28. Zantac		24 Brufen
	29. Epilim		
Supplementary	30. Zovirax	34. Diflucan	39. Crestor^b^
	31. Tegretol	45. Lenamet	43. Erythrocin
	32. Augmentin		
	33. Lanoxin		
	35. Nizoral		
	36. Cozaar		
	37. Epanutin		
	38. Rimactane		
	40. Tarivid		
	41. Aminophylline		
	42. Daktarin		
	44. Zithromax		
	46. Zestril		
	47. Clarityne		
	48. Fortum		
	49. Ismo		
	50. Eltroxin		
	30. Zovirax		
	31. Tegretol		
	32. Augmentin		
	33. Lanoxin		
	35. Nizoral		
	36. Cozaar		
	37. Epanutin		
	38. Rimactane		
	40. Tarivid		

^a^Change in dosage form

^b^Manufactured in 2006

### Trend 1

**Fig. 4:**
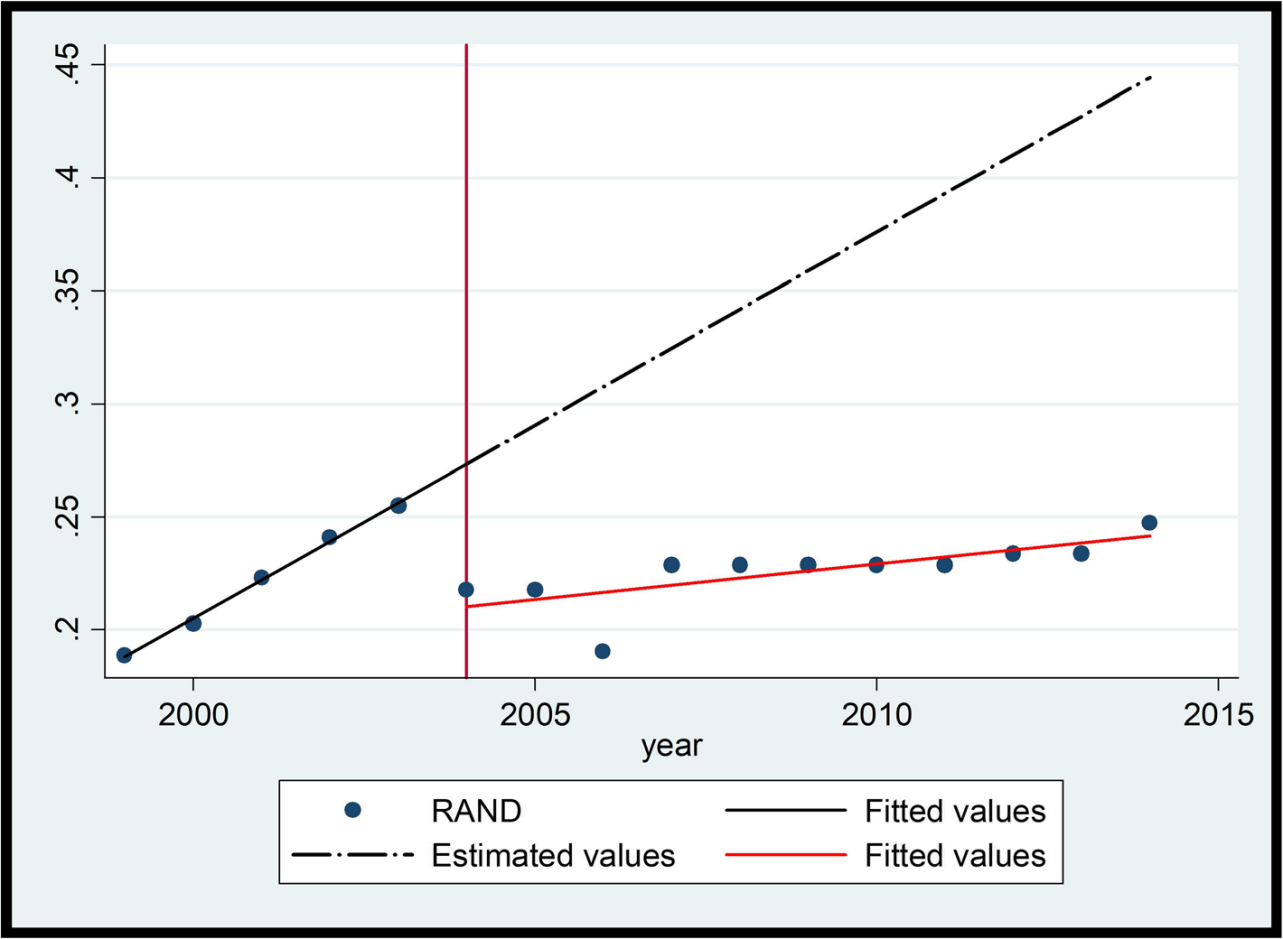
Ventolin® (Salbutamol 2 mg/5 ml) Syrup

Between 1999 and 2004, prior to the intervention, these medicines showed a significant year-on-year increase in price. Upon introduction of the intervention the medicines showed an immediate drop in price with a subsequent rate of increase being much less than before. Salbutamol (Fig. 4 and Table 5) will be used to illustrate the changes.

**Table 5:** Changes in levels and slopes of the three medicines illustrating the three trends observed

	Change in Level (*P*-Value)	95% Conf. Interval	Change in Slope (*P*-value)	95% Conf. Interval
Trend 1				
Salbutamol 2 mg/5mls Syr 1. Ventolin	−0.065 (0.000)	−0.085 - -0.046	−0.014 (0.000)	−0.02 - -0.009
Trend 2				
Ciprofloxacin 500 mg Tabs 7. Ciprobay	−5.113 (0.000)	−7.188 - -3.038	1.560 (0.000)	0.996–2.124
Trend 3				
Amitriptyline 25 mg Tabs 6. Tryptanol	−0.397 (0.000)	− 0.541 - -0.252	−0.169 (0.000)	− 0.208- -0.130

A visual inspection of the interrupted time series graph for Ventolin® above indicates that the medicine prices prior to 2004 showed a year-on-year steady rate of increase (slope 0.018 (*P* = 0.000) [CI 95% (0.012 - − 0.023)]. The introduction of the single exit price (SEP) regulations in 2004 saw a price reduction as indicated by the change in level − 0.065 (P = 0.000) [CI 95% (− 0.085 - − 0.046)]. In addition, the average rate of increase before the regulation was higher than the average rate of increase after the regulation as indicated in the change in slope of − 0.014 (P = 0.000) [CI 95% (− 0.02–0.009)].

The Adjusted R-Squared for Ventolin® is relatively high at 93.54% indicating that the fitted value closely correlates to the observed prices. The *P*-Value is 0.000 indicating that there is a probability of a significant difference in price of the medicine after the policy intervention.

### Trend 2

**Fig. 5:**
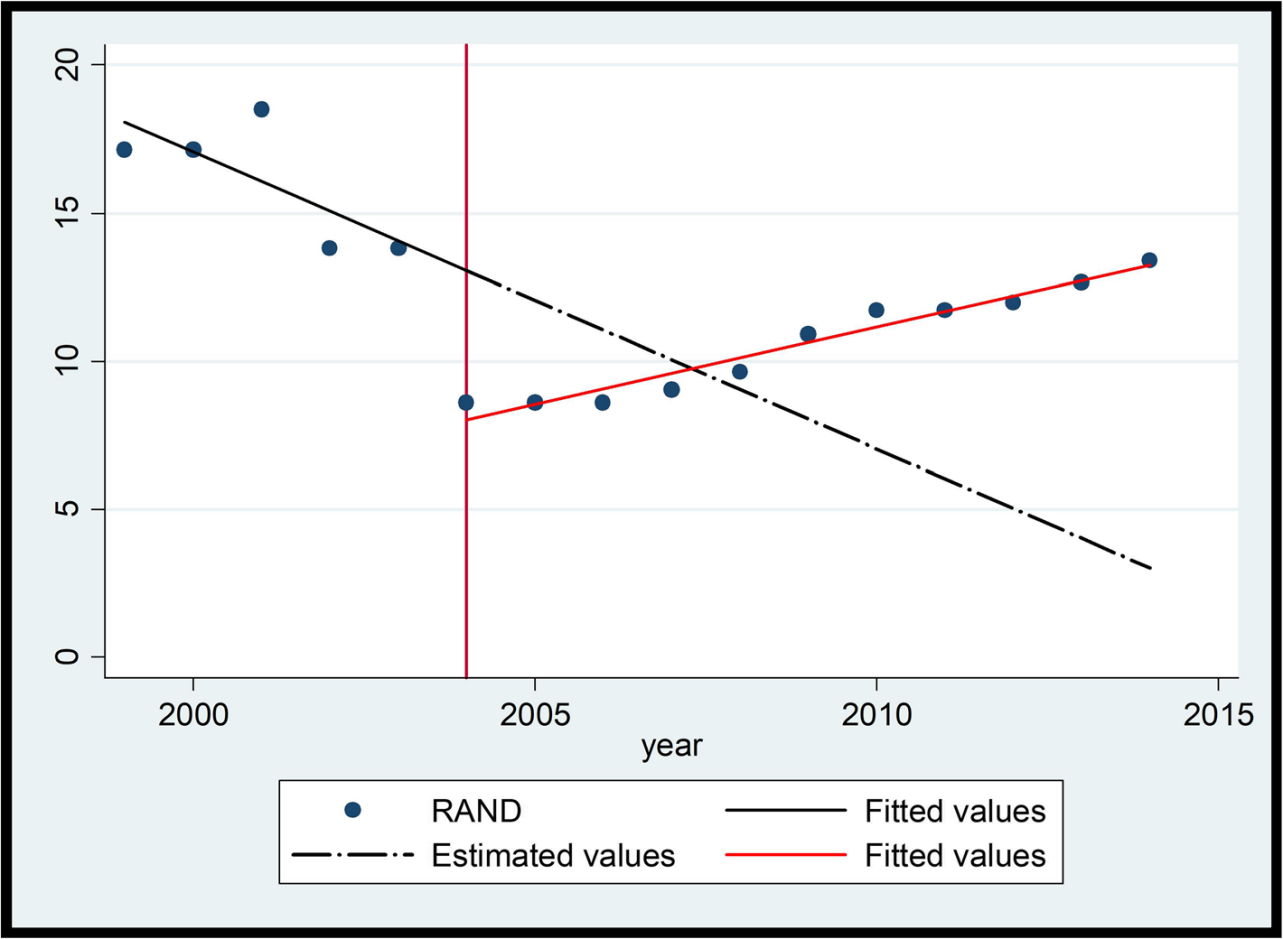
Ciprobay® (Ciprofloxacin 500 mg) Tablets

In trend 2 medicine prices were already decreasing prior to the intervention in 2004 as is evident in the visual inspection with Ciprobay® 500 mg (see Table 5). The average rate of decrease before intervention of Ciprobay® was ZAR 1.028 per year (*P* = 0.002) [CI 95% (− 1.579 - -0.478)] reflected in the slope. After intervention the medicine saw a price reduction as indicated by the change in level of − 5.113 (*P* = 0.000) [CI 95% (− 7.188 - -3.038)]. The average price increase after the introduction of the intervention in 2004 as opposed to a decrease is reflected in the change in slope of ZAR 1.560(P = 0.000) [CI 95% (0.996–2.124)]. The slope change in Trend 2 indicates that the medicines will lose most of their gains over time (see Fig. 5).

### Trend 3

Trend 3 is reflective of medicines that were withdrawn between 4 and 9 years after the introduction of the SEP regulations. There was a small number of medicines (11) in this basket, and the trend needs to be interpreted with care. Most (8 of 11) medicines showed overall price decrease of between 0.89–82.16% from 2004 until their withdrawal.

**Fig. 6:**
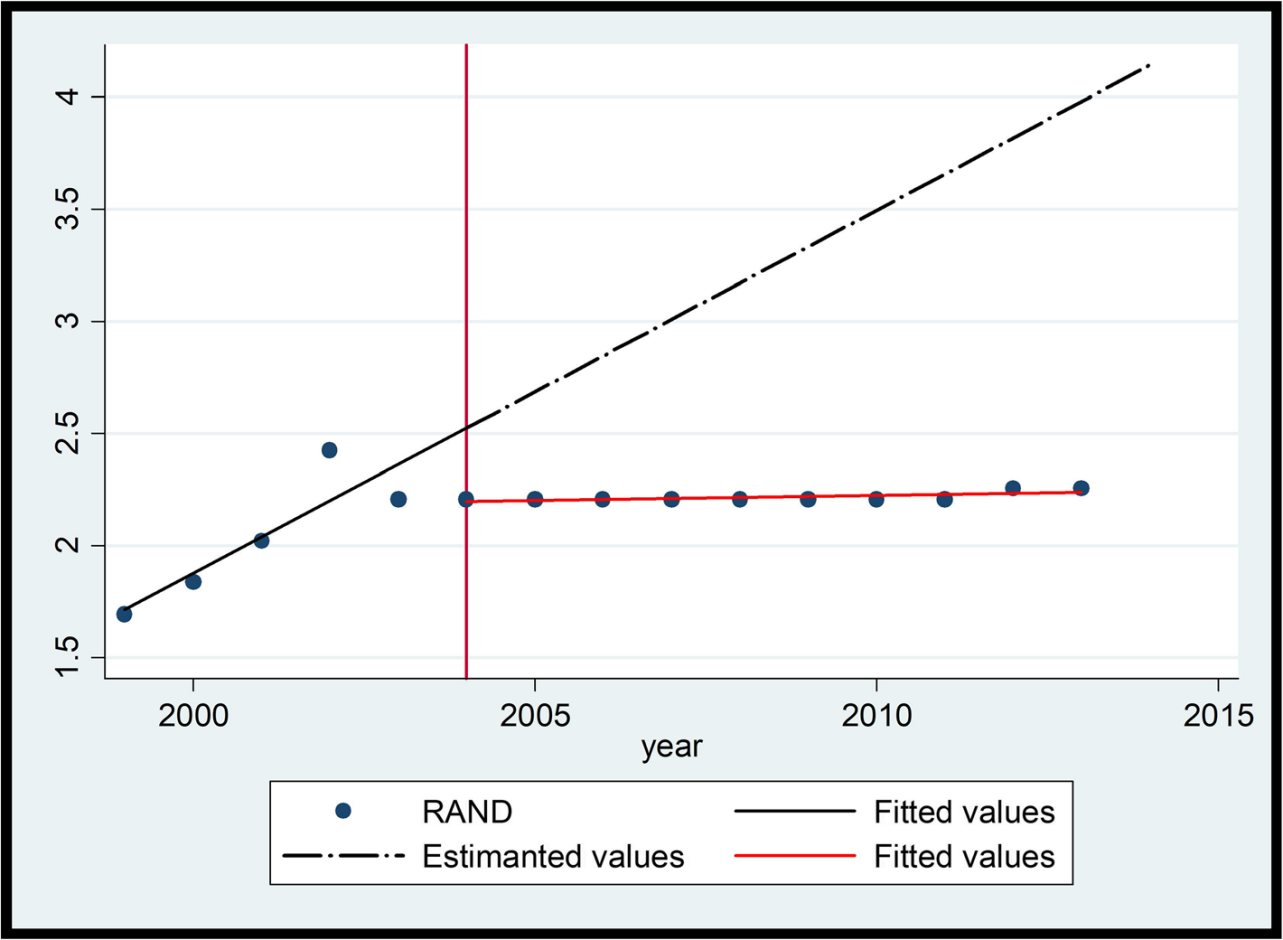
Tryptanol® (Amitriptyline 25 mg) Tablets

Trend 3 is illustrated using Tryptanol® 5 mg tablet (see Fig. 6 and Table 5). The Adjusted R-Squared for Tryptanol® 5 mg tablet is 98.79%. The *P*-Value is 0.000 indicating that there is a probability of a significant difference in price of the medicine after the policy intervention. The price reduction of the medicine due to the introduction of the intervention in 2004 is reflected in the change in level − 0.397 (*P* = 0.000) [CI 95% (− 0.541 - -0.252)] and the change in slope ZAR 0.169 (P = 0.000) [CI 95% (− 0.208–0130)].

### Medicines not subjected to SEP

One of the medicines of interest in the study was Paracetamol (Panado®) syrup. Paracetamol appeared on the list suggested by HAI and WHO in the Global Core and was therefore included but not subjected to the SEP (as it is schedule zero in South Africa and these medicines are exempt from pricing regulations). While the medicine showed an immediate 15% decrease in price in 2004 the price increased by 536% by 2014 as compared to the estimated value (see Table 6). If the medicine were subjected to the normal increase of SEP as determined and published by the National Department of Health Paracetamol (Panado®) Syrup would have been priced 18% less to the consumer today.

**Table 6:** Price trend for Paracetamol (Panado®) Syrup from 2004 to 2014

SEP Increases (%)	Year	Actual	Price with SEP	Diff bet Actual and SEP	% Increase
0	2004	0.16	0.16	0	0
0	2005	0.17	0.16	0.01	4.69
5.20	2006	0.18	0.17	0.01	5.84
0.00	2007	0.19	0.17	0.02	10.76
6.5	2008	0.21	0.18	0.03	12.46
3.2	2009	0.22	0.21	0.02	8.24
7.4	2010	0.24	0.23	0.00	1.06
0	2011	0.26	0.23	0.03	9.72
2.14	2012	0.27	0.24	0.04	12.98
5.8	2013	0.28	0.25	0.04	12.29
5.82	2014	0.31	0.27	0.05	15.26

## Discussion

This study of 50 originator medicines evaluated the legislated price control on the exit price of medicines in South Africa, a low-to middle-income country. Majority of the medicines investigated showed an immediate reduction in price in 2004. Moreno–Torres [4], looked at measures of price regulation in Spain over time. These interventions include reference pricing, mark-up reduction of wholesale distributors’ and retailers’ fees and compulsory reductions of ex-factory manufacturer prices. The results of the study [4] indicated that there was a negative impact on expenditure per capita, that was significant, by four of the interventions, while seven interventions with a negative impact on price and one with a positive impact on price. Three interventions had a positive impact on the number of prescriptions per capita (only one resulted in a reduction). This study indicates that the SEP regulation had a major impact on medicine pricing in South Africa in both the short and long term. Most medicines investigated showed a smaller yearly increase in price compared to before regulations due to the controlled pricing environment introduced by Government. Each year a stringent process exists where manufacturers apply for price increases through the established Pricing Committee and can only increase their medicines after the Minister of Health publishes an endorsed increase in medicine pricing (Regulation 8) [26]. The regulation also allows, under exceptional circumstances, for the Minister to approve increases as contemplated in Regulation 9 of the Medicines and Related Substances Act [26] taking into consideration the unintended consequences of business viability as an example. The results show that where there was a lower increase (slope) compared to prior to regulations the patients will continue to benefit from the regulations, a concept discussed in Sood et.al [5] where they concluded that the impact of price control measures on cost reduction increased the longer they remained in effect.

Further studies need to be done to determine availability and access [27] and possible negative impact of this type of pricing model. In addition, manufacturers currently determine their own costs, which may provide a potential risk to transparency. The previous stated intention to introduce international bench marking by government may overcome this potential threat. The South African policy may provide sufficient security to this risk in section 9 of the Medicines Act [26].

Those medicines in the study that reduced their prices prior to the introduction of the regulations (Trend 2) also showed a further saving in the 2004 period but lost this advantage as the manufacturers tended to take the annual price increases offered by Government. Further investigation is needed to understand why certain medicines decreased their prices even before the Government intervention. It may have been due to these medicines coming of patent, the introduction of generics or companies preparing for the expected price reduction as a business strategy so that a large sudden drop in the price did not adversely impact their market.

Of concern are the 16% (8 of 50) medicines that were withdrawn from the South African Market. One of the overarching policy considerations of the WHO/HAI Policy [25] document suggested that the policy choice should not undermine/impact a reliable supply of quality products. In the case of South Africa each of these medicines that were withdrawn had adequate supplies of quality generics available.

Their withdrawal therefore may have been as a result of competitive pricing of the generics; introduction of new generics or a business decision related to the subsequent non-profitability of the said medicine items to the manufacturer. Marie–Paule Kieny, WHO Assistant Director General for Health Systems and Innovation suggested that “When low prices preclude profits, companies leave the market – and leave a hole in the availability of quality products” [28]. It would be valuable to investigate all withdrawn molecules since 2004 and conduct an in-depth study to determine reasons for same.

The introduction of the pricing regulations (SEP) in South Africa created an ideal platform for pricing transparency, a concept that Vogler [29] agreed can contribute to affordable patient access to medicines. Clearly, the intervention showed a substantial decrease in medicine prices with most medicines showing a continued gain because of the controlled nature of the subsequent annual increases. The findings in this study concur with the conclusion previously articulated by Sood et.al [5], that the longer price control remained in effect, the greater the impact on cost reduction.

Controlling medicine prices at the manufacturer level is a common strategy in price control policies seen in most European Union countries [30] where authorities set the price on a regulatory basis. South Africa’s policy to do the same is thus in line with international practices. Internal reference pricing, international benchmarking, maximum prices, index pricing, price negotiations and volume based pricing are common pricing intervention methods used by various countries. A Cochrane review of the effects of pharmaceutical pricing and purchasing policies on health outcomes, healthcare utilization, drug expenditure and medicine use [30], included 18 studies in their main results, 17 of reference pricing (one included maximum pricing) and one of index pricing. The authors concluded that reference pricing may reduce relative expenditure on reference drugs but could not conclude on the shift to cost sharing with patients. The effects of other pricing policies studied in the review were uncertain due to sparse evidence and the authors concluded that studies needed to be spread to include low to middle-income countries. This study thus tries to add to the body of literature on pricing policies other than reference pricing, and from low and middle-income countries.

In the WHO Guidelines on Country Pharmaceutical Pricing Policies [3], a panel of experts recognized that the quality of research and evidence in relation to pharmaceutical policy implementation in developing countries was poor. South Africa adopted some of the key recommendations found in this policy document around medicine pricing for the private sector. Added to this the South African Government introduced control on the supply chain towards the retail price with the introduction of the regulated dispensing fee and a proposal to regulate distribution fee in the wholesale environment.

Certain limitations of this study must be taken into account. The first is the limited data available prior to implementation of the regulations. Bernal et.al [31] suggest that there are “no fixed limits regarding the number of data points”. The power depends on “various other factors, including distribution of data points before and after the intervention, variability within the data, strength of effect, and the presence of confounding effects such as seasonality” [30].

In inspecting the visual results, which is a recommendation by Bernal et.al [31], it can be seen that the trend before intervention does not show drastic changes. There is also a clear differentiation between the pre- and the post-intervention period with a well-defined period of implementation- in this case an immediate change [30].

The authors note the nonexistence of a control as a further limitation. Using the same selected medicines in the public sector was not possible as the state is subject to a tender process and the price data is limited. The state is also undergoing its own reform in the form of STGs, EML and class tenders. In this case while it may be possible to use non-equivalent control as suggested by Penfold [32] this did not exist.

A further limitation is acknowledged in the price files collected from the vendor supplying pharmacies. However, these price files are derived from the SEP database and organized in terms of electronic format for pharmacy use. Thus all pharmacies are reliant on these price and data files. The company has a track record of more than 20 year and supplies these price files to more than 65% of the industry. The SEP is further checked at pharmacy level when claims are submitted to payers for verification.

The study evaluates the impact of the SEP on a basket of original medicines, in terms of costs, immediate price reductions and projected price reductions. The authors acknowledge the limitation that a change in medicine price determines change in expenses but it doesn’t imply savings. This could be the subject of further research.

The last limitation is the linear trend assumed by the segmented regression model that was used [22]. Despite these, this study provides evidence of the impact of medicine pricing intervention from a middle–income country, and useful lessons can be drawn by other developing countries looking at introducing medicine price controls.

## Conclusion

South Africa embarked on attempting to reduce medicine prices through SEP. This study attempted to quantify the impact of the Single Exit Price (SEP) regulation.

The research conducted here confirms that substantial price reductions have been achieved through the introduction of the SEP regulation, despite the fact that other research in this field suggests that single interventions may not be sufficient in delivering affordable, accessible medicine.

## Data Availability

The data file can be made available on request to the authors.

## Abbreviations

CMS: Council for Medical Schemes
COMED: Central Procurement Agency for medicines in South Africa
EML: Essential Medicine List
ERP: External Reference Price
HAI: Health Action International
IRP: Internal Reference Price
ITS: Interrupted Time Series
LMICs: low to middle-income countries
MCC: Medicine Control Council
NAPPI: National Pharmaceutical Product Index
NDP: National Drug Policy
NHA: National Health Accounts
NHI: National Health Insurance
No-POS: Complimentary Health Plan of Colombia
OECD: Organisation for Economic Co-operation and Development
POS: Compulsory Health Plan of Colombia
SEP: Single Exit Price
STGs: Standard Treatment Guidelines
UHC: Universal Health Coverage
WHO: World Health Organisation
ZAR: South African Rand
